# Simultaneous Influence of Gradients in Natural Organic Matter and Abiotic Parameters on the Behavior of Silver Nanoparticles in the Transition Zone from Freshwater to Saltwater Environments

**DOI:** 10.3390/nano12020296

**Published:** 2022-01-17

**Authors:** Ivana Čarapar, Lara Jurković, Dijana Pavičić-Hamer, Bojan Hamer, Daniel Mark Lyons

**Affiliations:** Center for Marine Research, Ruđer Bošković Institute, G. Paliaga 5, 52210 Rovinj, Croatia; ivana.hazdovac@irb.hr (I.Č.); lara.jurkovic@irb.hr (L.J.); dpavicic@irb.hr (D.P.-H.); bhamer@irb.hr (B.H.)

**Keywords:** estuary, brackish water, urban wastewater, dissolved organic carbon, biocorona, agglomeration, kinetics

## Abstract

As nanoparticles have been found to cause a range of harmful impacts in biota, understanding processes and transformations which may stabilize and increase their persistence time in the environment are of great importance. As nanoparticles carried in riverine or wastewaters will eventually reach estuaries, understanding their behavior and transport potential in this transition zone from fresh to marine waters is essential, particularly as estuaries are sensitive ecological zones, oftentimes encompassing ornithologically important areas. In this direction, we report on the influence of combined gradients of riverine and marine natural organic matter (NOM) on the temporal stability of biocorona-encapsulated silver nanoparticles in terms of ion release kinetics. In parallel, salinity, pH and oxygen saturation were simultaneously varied to create a model to mimic the complex estuarine environment. While humic acid (HA) and alginate (Alg) disrupted the stabilizing ability of the nanoparticle protein corona to a greater and lesser degree, respectively, they slowed the rate of ion release in freshwater at pH 6.6 and in saltwater at pH 8, respectively, while oxygen saturation was also found to be an important factor. Thus, as the type of NOM changes with pH along a salinity gradient in an estuary, conditions required to increase the persistence time of nanoparticles are serendipitously met, with greater colloidal stability achieved in cases where there is more rapid replacement of HA with Alg. Despite the strong gradients in ionic strength, pH and oxygen saturation, the protein corona was not sufficiently disrupted at the nanoparticle surface to be substituted by NOM indicating the greater adsorption energy of the protein’s hydrophobic domains. Ultimately, it is the specific NOM profile of individual estuaries that may provide the best indicator for predicting the stability and persistence of silver nanoparticles as they transition from fresh to salt water environments.

## 1. Introduction

Research in the area of nanoscale materials has continued to grow apace over the past two decades. In parallel, the range of nanomaterials which have found applications in a wide variety of consumer and industrial products is also rapidly continuing to increase. Such use, deriving from their specific size-related physical and chemical properties, spans a broad array of products from textiles and detergents to pesticides, paints, and consumer electronics [1,2,3]. Indeed, there are currently over 3000 nanomaterial-containing products on the market, as listed at the online Nanodatabase (http://nanodb.dk/en/, accessed on 30 October 2021).

However, with increasing production and use of nanomaterials, it has become clear over the past number of years that the potential for release of such materials into the environment, either accidently or by design, is significant. While the fate of nanomaterials in the environment still remains to be fully understood, advances are being made in understanding their physico-chemical behavior in various environmental compartments and how such materials may be transformed and transported, in particular in freshwater aquatic systems [4,5].

Among nanoparticles, the potential for silver nanoparticles to reach the environment has received much attention not only because of silver nanoparticles’ widespread use, but also because of their biocidal properties. For example, use of silver nanoparticles in textiles or in personal care products due to their antibacterial properties has led to their release into urban wastewater streams [6,7,8]. While such streams may be directed to wastewater treatment plants (WWTP) where silver nanoparticles can undergo rapid dissolution in the sulphur-rich environment, storm surges may result in their escape from WWTP before complete deactivation [9]. In addition, wastewater streams oftentimes end in outflows to estuaries or coastal waters rather than being diverted to WWTP, thus representing a more direct route for silver nanoparticles to enter freshwater, brackish and marine waters [10].

Silver nanoparticles gradually undergo oxidative dissolution in aquatic systems [11]. In oxic conditions, an Ag_2_O layer forms on the nanoparticle surface which gradually dissolves, subsequently exposing underlying silver to oxidation. This is a thermodynamically favorable process which eventually leads to complete dissolution of the nanoparticle. The kinetics of this oxidation are enhanced as the size of the nanoparticle decreases and at lower pH, indicating the facilitating role of H^+^ in the process. However, it should be noted that the presence of sulphur, for example from dissolved organic matter, also contributes to the oxidation of silver nanoparticles, forming the corresponding sulphide (Ag_2_S). While such oxidation pathways are more typical for silver nanoparticles in wastewater streams, wastewater treatment plants and freshwater, in brackish and seawater the additional presence of high concentrations of Cl^−^ ions presents a further factor in nanoparticle oxidation. Specifically, a range of soluble chloride species may form, including AgCl, AgCl_2_^−^ as well as lesser quantities of AgCl_3_^2−^ and AgCl_4_^3−^ species, thus providing an additional pathway to silver oxidation. However, competing silver reduction processes have also been noted. For example, immediately after addition of silver nanoparticles to NaCl solution (at concentrations up to about 150 mM) the formation of new silver nanoparticles was noted and ascribed to the reduction of silver ions present [12]. Indeed, it has been known for some time that in natural waters similar silver ion reduction may occur due to the action of sunlight in the presence of dissolved organic matter such as humic acid [13,14] and fulvic acid [15]. Furthermore, greater silver reduction was found for relatively low dissolved carbon concentrations (2.4 mg-C L^−1^) in river waters compared to humic acid test solutions of 4 mg-C L^−1^, suggesting that other factors apart from light intensity also play a role [16].

Apart from modulating oxidative dissolution and/or the re-formation of silver nanoparticles by reduction of released ions, natural organic matter (NOM) present in natural waters and wastewater streams also plays an important role in controlling the aggregation and dissolution of nanoparticles. In particular, it has been noted that various types of interaction can lead to significantly different aggregation outcomes, especially in waters where electrolyte concentrations are high. Considering that NOM is typically negatively charged, interaction with electropositive silver nanoparticles may proceed by electrostatic attraction (usually for smaller molecules), chelation, steric stabilization due to the relatively large size of some NOM or an electro-steric combination of the two, with the adsorbate with the highest adsorption energy outcompeting other molecules present including capping ligands on the nanoparticles. The nature of the interaction of NOM with the nanoparticle surface also varies as a function of pH, with binding of carboxyl groups noted at lower pH values characteristic of freshwaters while aliphatic moieties and phenol groups were more likely to bind, particularly through hydrogen bonding, at alkaline pHs more typical of marine waters [17]. It has been reported that greater colloidal stabilization from NOM proceeds in the order bovine serum albumin > humic acid > fulvic acid > alginate, with the larger molecules providing more efficient coverage and binding to the surface [18]. Moreover, it was noted that at higher divalent cation concentrations, colloidal destabilization occurred irrespective of the type of NOM present. On the contrary, a study reported that bovine serum albumin can stabilize silver nanoparticles in artificial seawater of salinity S·38 (electrolyte concentration ~0.7 M) for up to two weeks [19]. Similarly, it was found that natural polysaccharides such as alginate or gum Arabic greatly slow nanoparticle aggregation in fjord water (salinity S·35), with half the surface plasmon resonance peak area still remaining after 5 h, compared to a solution of only NaCl at the same ionic strength [12]. While the stabilizing ability of NOM is now well known, particularly in low-strength electrolytes, the situation in high salt content media such as seawater is more complex. Apart from compressing the electric double layer around nanoparticles resulting in reduced repulsive forces (hence favoring agglomeration), divalent cations such as Ca^2+^ [20,21] or solid-phase AgCl [22] have also been found to act to bridge NOM between nanoparticles, for example through carboxylate functional groups and conformational changes in humic acids, which acts to enhance aggregation [23]. Ultimately, NOM-based stabilization of nanoparticles against aggregation not only affects dissolution and ion release processes but potentially increases nanoparticle persistence time in the water column which results in their being more available to biota [19]. This is particularly important, as the released ions have generally been found to be the primary drivers of silver nanoparticle toxicity to biota [24,25,26]. For example, by forming a passivating coat on silver nanoparticles through sulfidation, release of Ag^+^ ions was reduced with a corresponding reduction in nanoparticle toxicity to the bacterium *Escherichia coli* [27]. It is, therefore, clear that the behavior of silver nanoparticles in the aquatic environment is complex and dynamic, with a wide range of factors influencing their fate, which also encompasses their interaction with biota.

In estuaries, brackish water represents a highly complex transition zone between freshwater and saltwater, with strong gradients of NOM in terms of both concentration and composition and large variations in abiotic parameters including salinity, pH, light intensity, and temperature [28,29,30,31]. Likely due to this complexity, the behavior of nanoparticles in brackish waters has not received significant attention to date. However, estuaries represent sensitive ecological areas and, due to silt deposition and presence of mud flats, are also oftentimes protected as ornithological reserves. Thus, the potential for any harmful material of anthropogenic origin such as silver nanoparticles to persist in such areas is a cause for concern and requires investigation. However, reports in the literature on the behavior of silver nanoparticles in aquatic matrices typically focus on variations of individual parameters such as salinity or NOM concentration in isolation, and more realistic data on the fate of silver nanoparticles when multiple parameters are varied simultaneously are lacking. In this direction, the present study reports on the physico-chemical behavior of silver nanoparticles in a multi-parameter model simultaneously encompassing gradients of NOM and the primary abiotic factors of estuarine environments with a view to gaining greater understanding of the fate of these nanomaterials in brackish waters. In particular, the study takes a stepwise approach to resolving the behavior of silver nanoparticles, firstly in the presence of single or multiple types of NOM as a function of salinity and time. The study extends this by probing the effect of pH changes (simulating fresh, brackish and sea waters) on the behavior of the previously identified most stable multiple organic matter-coated silver nanoparticles. As a third step, oxygen saturation of the aqueous matrices in the prior experiment is reduced to determine the role of dissolved oxygen on silver nanoparticle fate as a function of salinity, natural organic matter, and pH. This approach provides systematic data on how multiple abiotic parameters and combinations of NOM simultaneously influence silver nanoparticle behavior as they transition from fresh to salt water in estuaries.

## 2. Experimental

Citrate-stabilized colloidal silver nanoparticles (AgNP) of diameter 40 nm (20 mg L^−1^, aqueous dispersion) were purchased from nanoComposix (San Diego, CA, USA). Sodium chloride, sodium hydroxide, potassium chloride, calcium chloride, magnesium chloride hexahydrate, magnesium sulfate heptahdrate, humic acid sodium salt (HA), sodium alginate from brown algae (Alg) and bovine serum albumin (BSA) were purchased from Sigma–Aldrich (MilliporeSigma, St. Louis, MO, USA) and used without further purification. Ultrapure water (18 MΩ·cm; mQ) was provided by a Millipore Advantage System (MilliporeSigma, Burlington, MA, USA).

Artificial seawater (ASW) was prepared by dissolving inorganic salts in mQ water in the following amounts: NaCl (450 mmol), KCl (10 mmol), CaCl_2_ (9 mmol), MgCl_2_∙6H_2_O (30 mmol) and MgSO_4_∙7H_2_O (16 mmol). The volume of mQ added was varied so as to achieve the desired salinity, e.g., 1 L mQ for an ASW stock solution of salinity S∙38. ASW of S∙10 and S∙20 were prepared by diluting the S∙38 stock solution with appropriate quantities of mQ water. Stock solutions of HA and Alg were prepared at a concentration of 100 mg L^−1^, while BSA stock solution was prepared at a concentration of 300 mg L^−1^. These stock solutions were further diluted such that the final NOM concentrations in the samples being measured were in the range 0.01–10 mg L^−1^ for HA and Alg, while BSA concentrations were in the range 0.1–100 mg L^−1^. These concentrations encompass those which may be encountered in fresh-, salt- and wastewater systems.

Sample preparation typically comprised of mixing AgNP with organic matter and then adding ASW to give the required salinity; for example, 75 µL AgNP dispersion was vortexed with 125 µL organic matter solution (HA, Alg, BSA or combinations thereof) in mQ water to which was then added 100 µL ASW of appropriate concentration. The final volume in microplate wells was always 300 μL, AgNP concentration 5 mg L^−1^ and NOM concentrations were in the ranges as indicated above. All solutions were freshly prepared before each experiment. Experiments were conducted at 25 °C and samples were monitored over a period of 4 days.

The influence of pH on colloidal stability over time at different salinities (S∙0–38) was determined at three pH values: 6.6, 7.2 and 7.8. Samples were prepared in the same way as previously by sequentially mixing AgNP with NOM and ASW, but using 10x greater volumes. The pH of these samples (3 mL) was adjusted using NaOH and monitored by Mettler Toledo SevenCompact S220 pH meter (Greifensee, Switzerland) and microelectrode, with 300 μL aliquots subsequently pipetted into microplate wells for measurement. Samples were held under nitrogen between measurements (Labconco glovebox, Kansas City, MO, USA) to avoid CO_2_ absorption and consequent change in pH.

To determine the effects of dissolved oxygen on AgNP behavior at different salinities (S∙0–38), a parallel series of samples (3 mL per sample) were prepared in the same way as before with NOM and mQ or ASW being added to AgNP followed by pH adjustment; however, the mQ or ASW had been boiled and cooled under nitrogen purge to reduce the dissolved oxygen concentration. Water thus treated showed oxygen saturation of approximately 50–60% while water in air had a saturation level of ~90%. Oxygen concentration was monitored by a Hanna Instruments HI 9146 (Woonsocket, RI, USA) dissolved oxygen meter, and samples were held under nitrogen in a glovebox, except when measurements were being conducted, to avoid uptake of oxygen.

The surface plasmon resonance of AgNP, as a proxy for colloidal stability, was monitored by absorption spectroscopy. Spectra were recorded for samples in 96-well clear polystyrene microplates on a Tecan Infinite M200 Pro plate-reader (Männedorf, Switzerland) in the wavelength range 280–1000 nm, and for samples in 1 cm path length quartz cuvettes on a double-beam Shimadzu UV-1800 spectrophotometer (Kyoto, Japan) in the wavelength range 190–1100 nm. Data were processed with proprietary Tecan Magellan, version 7.2 (Tecan Austria GmbH: Grödig, Austria, 2016) and Shimadzu UVProbe, version 2.31 software (Shimadzu Corporation: Kyoto, Japan, 2007), respectively. Silver ion concentrations were measured by inductively coupled plasma mass spectrometry (ICPMS) on an Element 2 instrument (Thermo Fisher Scientific, Waltham, MA, USA) after centrifugal ultrafiltration of nanoparticle dispersions through a 3 kDa molecular weight cut-off PES membrane (Sartorius Vivaspin 500, Göttingen, Germany). Statistical analysis was carried out using OriginPro, version 9.0 (OriginLab Corporation: Northampton, MA, USA, 2012).

## 3. Results and Discussion

Gaining an understanding of the processes that influence AgNP dissolution in the environment is particularly important due to silver ions’ well-known properties as strong antibacterials and antimicrobials. Silver nanoparticles undergo oxidative dissolution over time in all environmental matrices, e.g., natural waters, sediment and soil, particularly those that have high oxygen or sulphur content [8]. The kinetics of such dissolution may be modulated by natural organic matter, either through formation of a corona around the nanoparticles or by influencing agglomeration and aggregation processes, and hence the surface area of the nanoparticles in contact with the surrounding medium. For example, Suwannee River humic acid and fulvic acid in the range 1–50 mg L^−1^ were found to reduce the release of Ag^+^ ions from 5 nm diameter nanoparticles in a concentration dependent manner, and ion release in seawater over the course of 24 h was about 20% of the total AgNP mass, although pH was found to have the greatest influence on the dissolution process [28]. Furthermore, electrolyte strength plays a critical role, where increasing salinity acts to disrupt the electrical double layer around nanoparticles, promoting agglomeration [29]. However, it should be noted that competing processes where released ionic silver may be reduced to zero-valent silver under the influence of sunlight and environmentally relevant concentrations of NOM such as Suwannee River fulvic acid, for example over a period of 8 h, can also play a role in the complex dynamics of such systems [32]. Environmentally (UV) aged AgNP were also found to behave differently from freshly prepared AgNP, where the former showed greater attachment to sand, but were more readily dissolved [33]. In that study, AgNP with smaller diameters (10 nm) than those used in the present work were found to release up to 30% of their mass as Ag^+^ ions over 7 days. As may be expected, light intensity was also found to be an important factor, with significant photoreduction of Ag^+^ ions noted after just 1 h in the presence of environmentally relevant NOM concentrations of ≤5.5 mg-C L^−1^ [16]. Ultimately, all of these competing factors which govern silver nanoparticle dissolution in aquatic matrices become even more complex in estuarine brackish waters due to the type and concentration of the NOM present which varies from the lower reaches of rivers to open coastal waters. Moreover, strong gradients in salinity, pH, oxygen saturation, temperature and light transmission in the water, which all concomitantly vary, highlights the challenge in attempting to resolve AgNP dissolution processes in the freshwater to seawater transition zone. As a step in this direction, four key variable parameters, viz NOM, salinity, pH and oxygen saturation, were investigated for their combined impact on AgNP dissolution.

HA was selected as a model of NOM in fresh and brackish water, consisting of a range of acidic organic polymers rich in carboxylic and phenolic groups that can be extracted from humic substances (humus) in soil, sediment or aquatic environments. Alg, chosen as a representative of NOM in seawater, is an anionic polysaccharide extracted from marine brown algae and contains 1-4′-linked β-D-mannuronic acid and α-L-guluronic acid residues of widely varying composition, while BSA was used as a model for proteinaceous matter present in organic-rich wastewater streams.

### 3.1. Behavior of AgNPs as a Function of NOM and Salinity

**Figure 1:**
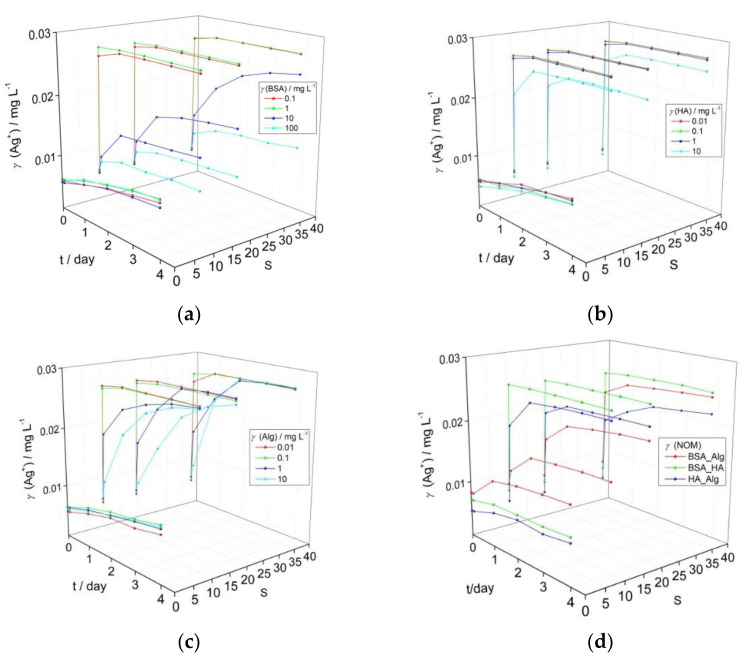
Temporal change in calculated Ag^+^ ion concentration based on intensity of the SPR peak maximum for AgNP coated with (**a**) bovine serum albumin (BSA), (**b**) humic acid (HA), (**c**) alginate (Alg), and (**d**) combination of natural organic matter (NOM; BSA, HA and Alg concentrations of 10, 10 and 1 mg L^−1^, respectively) as a function of salinity (S∙0–38). pH of the dispersions was 7.8.

The influence of BSA, HA and Alg as proxies for natural NOM present in wastewater streams, freshwater, and seawater, respectively, on AgNP dissolution is shown in Figure 1. Ag^+^ ion concentrations reported herein were calculated based on AgNP surface plasmon resonance (SPR) intensity, where preliminary experiments showed a linear relationship between SPR intensity and measured Ag^+^ ion concentration (Supplementary Figure S1). Typical UV-vis absorption spectra for AgNP incubated with NOM are given in supplementary information Figure S2. The temporal evolution of the AgNP surface plasmon resonance (SPR) peak maximum, and hence Ag^+^ ion release, shows distinctly different behavior as a function of salinity for BSA concentrations of 0.1–100 mg L^−1^ and HA and Alg concentrations in the range 0.01–10 mg L^−1^. These NOM concentrations were chosen as they broadly encompass the quantities that may be present in their respective environmental compartments, with, for example, humic substances present in higher quantities in upper estuaries with alginate increasing in concentration in lower estuaries and coastal waters.

BSA did not induce a strong reduction in the nanoparticle SPR in mQ over a period of four days for all tested concentrations, and calculated released Ag^+^ ion concentrations increased from 0.006 to 0.010 mg L^−1^ (Figure 1a). As the electrolyte concentration increased, only BSA in the 10–100 mg L^−1^ range enabled the SPR to remain over the investigated period (for example, temporal evolution of SPR for AgNP with 10 mg L^−1^ BSA over 4 days is shown in Supplementary Figure S3). For example, at the highest BSA concentration of 100 mg L^−1^ and after 4 days, the calculated Ag^+^ ion concentration increased to 0.010, 0.010 and 0.012 mg L^−1^ at salinities S·10, 20 and 38, respectively. Similarly, AgNP in the presence of HA at S∙0 (mQ) still maintained relatively good stability (Figure 1b) over four days, with an overall increase of Ag^+^ concentration to 0.010 mg L^−1^, irrespective of the HA concentration. With increasing salinity, an initial rapid reduction in the SPR absorbance likely indicated some agglomeration, with only the highest HA concentration of 10 mg L^−1^ showing a stabilizing effect as indicated by the continued presence of the SPR peak. However, a peak characteristic of AgNP agglomeration, typically centered at wavelengths of about 550 nm in the UV-vis absorption spectrum, did not appear as the SPR absorbance decreased [34,35]. Based on the decrease in SPR intensity, calculated Ag^+^ ion concentrations quickly increased to 0.027 mg L^−1^ at salinities of S·10, 20 and 38 for HA concentrations up to 1 mg L^−1^, while the highest HA concentration of 10 mg L^−1^ led to slower Ag^+^ ion release which reached 0.025 mg L^−1^ by day 4 in ASW S·38. Just as for the other types of NOM, Alg did not strongly disrupt colloidal stability in mQ, with calculated Ag^+^ ion concentrations increasing from initial values of 0.005–0.006 mg L^−1^ to reach 0.009–0.010 mg L^−1^ after 4 days. Alg-encapsulated AgNP showed behavior intermediate between BSA and HA in ASW where the SPR intensity gradually diminished over several days for Alg concentrations in the 1–10 mg L^−1^ range. The calculated Ag^+^ ion concentrations within a day rapidly increased to 0.027 mg L^−1^ for Alg concentrations of 0.01 and 0.1 mg L^−1^ at all ASW salinities while the samples with higher Alg concentrations of 1 and 10 mg L^−1^ showed ion concentrations that increased more slowly over the first day, reaching 0.022 and 0.019 mg L^−1^, respectively, in ASW S·10, with values of 0.022 and 0.022 mg L^−1^, respectively, noted in ASW S·38. Eventually, Ag^+^ ion concentration was calculated at about 0.027 mg L^−1^ after 4 days (Figure 1c).

The reason for BSA’s ability to stabilize AgNP to a greater extent than other NOM may be related to hydrophobic domains in the molecule which may orientate towards and form a relatively compact corona around the nanoparticles similar to salting-out-like behavior. A previous study showed excellent agreement between reduction of SPR and increase of silver ion concentration in dispersions of BSA-encapsulated AgNP in saltwater [19]. However, the capacity of BSA to sequester silver ions released from the nanoparticle was not determined, so the silver ion concentration measured after centrifugal ultrafiltration may have been somewhat lower than the quantity of silver ions actually released.

Based on the data herein, the lowest concentrations of NOM that indicated an ability to stabilize AgNP colloids and slow Ag^+^ ion release over 4 days under a range of salinities were selected for subsequent experiments. Thus, BSA, HA and Alg concentrations of 10, 10 and 1 mg L^−1^, respectively, were tested in combinations to determine if interaction between different types of organic matter may act to enhance or decrease colloid stability. This represents a more realistic scenario, as nanoparticles are unlikely to first encounter HA or Alg in the environment in a pristine state, e.g., as bare or citrate-coated nanoparticles, but will rather have already accumulated a bio(eco)corona as they pass through wastewater streams after release from, for example, laundered textiles and cosmetics. Thus, it is important to determine how AgNPs encapsulated in an eco-corona will behave when coming into contact with HA in fresh or brackish waters, and with Alg in brackish or coastal waters (Figure 1d).

While there were small increases in calculated Ag^+^ ion concentration in mQ over the investigated period, particularly for combinations of HA with BSA (0.001 mg L^−1^) or Alg (0.002 mg L^−1^), an increase in released ion concentration within the first day of 0.004 mg L^−1^ was noted for Alg added to BSA-encapsulated AgNP, suggesting some disruption of the stabilizing BSA coating on the nanoparticle surface. HA in low to medium salinity waters (S∙10–20) led to rapid increase of Ag^+^ ion concentration to values of about 0.018 mg L^−1^ indicating that it quickly and effectively disrupted the stabilizing ability of BSA at those electrolyte strengths (Figure 1a). For mid- to high-salinity waters (S∙20–38), when BSA-coated AgNP are more likely to encounter Alg rather than HA, Alg was found to stabilize AgNP-BSA to a greater degree than HA, with the AgNP SPR absorbance peak clearly evidenced up to four days after the addition of salt water, and calculated Ag^+^ ion concentrations reaching values of 0.020 and 0.024 mg L^−1^ at S·20 and 38, respectively. Interestingly, the interaction of Alg with HA-coated nanoparticles indicated greater stability with increasing salinity, and was the most stabilizing combination of NOM at S·38 with final ion concentrations of 0.022 mg L^−1^ calculated, i.e., 11–13% less than for the other NOM combinations). In summary, all types of NOM provide good AgNP stabilization in freshwater, but as salinity increases only BSA continues to stabilize the colloid, some stabilization is provided by Alg, while HA shows poor effectiveness. However, combinations of NOM at the nanoparticle surface show different behavior, with BSA-Alg proving superior to BSA-HA in stabilizing the colloid at all salinities.

### 3.2. Behavior of AgNPs as a Function of NOM Combinations, Salinity, and pH

**Figure 2:**
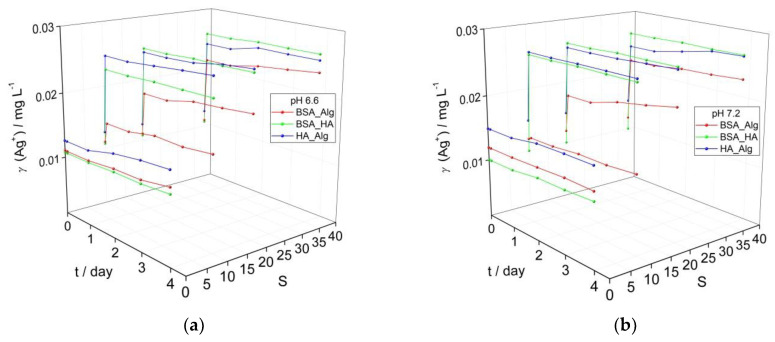
Temporal change in calculated Ag^+^ ion concentration based on intensity of the SPR peak maximum for AgNP coated with combinations of NOM (BSA, HA and Alg concentrations of 10, 10 and 1 mg L^−1^, respectively) at (**a**) pH 6.6, and (**b**) pH 7.2 as a function of salinity (S∙0–38).

While marine waters have alkaline pH values of about 8, surface waters such as rivers commonly have less alkaline pH in the range of 6–8. To investigate whether a gradient in pH can significantly affect the behavior of AgNP as they transition from freshwater to saltwater, the pH of AgNP dispersions coated with a combination of NOM was reduced from 7.8 (Figure 1d) to 7.2 and 6.6 (Figure 2) and the impact on the SPR was noted. The SPR-derived initial Ag^+^ ion concentrations of about 0.01 mg L^−1^ for AgNP treated sequentially with different combinations of NOM (Figure 2) are greater than the corresponding values (0.005 mg L^−1^) for AgNP treated with individual types of NOM (Figure 1). This is ascribed to the longer time required for incubating the AgNP with several types of NOM and adjusting the pH which results in a greater loss of SPR intensity (greater Ag^+^ ion release) before time measurements had started (i.e., the designated *t* = 0). The same trends were observed for all dispersions as a function of salinity and NOM, irrespective of pH. While dispersions showed little increase in calculated Ag^+^ ion concentrations over 4 days in mQ, placing AgNP in saltwater resulted in an immediate sharp decrease in SPR for those coated with BSA-HA and HA-Alg, indicating some agglomeration and likely increased ion release. For example, already after 1 day, the calculated Ag^+^ ion concentrations for the BSA-HA and HA-Alg combinations had increased to 0.023 and 0.025 mg L^−1^, respectively, in ASW S·10 and 0.025 mg L^−1^ in ASW S·38. The SPR, and hence the calculated Ag^+^ ion concentrations, remained relatively constant for the next 3 days. A smaller initial SPR decrease was noted for the corresponding BSA-Alg-treated nanoparticles and whose SPR absorbance also remained relatively constant to the end of the experiment, even at the highest salinity (S∙38). The calculated Ag^+^ ion concentrations increased to 0.015, 0.020 and 0.024 mg L^−1^ in ASW S·10, 20 and 38, respectively, at pH 6.6; 0.013, 0.021 and 0.023 mg L^−1^ in ASW S·10, 20 and 38, respectively, at pH 7.2; and 0.013, 0.019 and 0.024 mg L^−1^ in ASW S·10, 20 and 38, respectively, at pH 7.8, after 4 days. In summary, the BSA-HA combination provided slightly greater stability in mQ at lower pH values (pH 6.6, 7.2) which correlates with river waters just entering an estuary. As the salinity and pH increase, representing the transition to marine waters, only the presence of Alg was found to provide any significant stabilization of the BSA-coated AgNP and reduced Ag^+^ ion release, while HA was noted to be relatively ineffective.

### 3.3. Behavior of AgNPs as a Function of NOM Combinations, Salinity, pH and Oxygen Saturation

**Figure 3:**
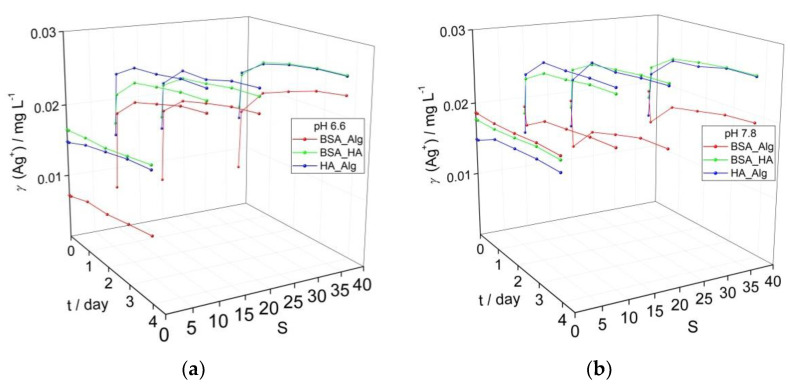
Evolution of calculated Ag^+^ ion concentration based on intensity of the SPR absorbance in media with low oxygen saturation (50%) for dispersions of AgNP coated with combinations of NOM (BSA, HA and Alg concentrations of 10, 10 and 1 mg L^−1^, respectively) at (**a**) pH 6.6, and (**b**) pH 7.8 as a function of salinity (S∙0–38).

In contrast to NOM-encapsulated AgNP in oxygen-saturated waters at pH 6.6 (Figure 2a) and pH 7.8 (Figure 1d), AgNP dispersed in solutions with reduced oxygen saturation levels of about 50% showed slower rates of SPR absorption peak reduction over time at both pH 6.6 and pH 7.8, ascribed to slower Ag^+^ ion release (Figure 3). In particular, BSA-HA and HA-Alg showed higher SPR intensities at pH 6.6, while BSA-Alg showed high stability at all salinities at pH 7.8. As oxidative dissolution of AgNP is expected to primarily progress by the reaction:

2Ag_(s)_ + ½ O_2(aq)_ + 2H^+^_(aq)_ ↔ 2Ag^+^_(aq)_ + H_2_O_(l)_ (1)

both oxygen saturation and pH are key parameters. The slower reduction of the SPR peak at lower oxygen saturation observed herein, and the small difference in SPR peak intensity for corresponding samples at pH 6.6 and 7.8, indicates that oxygen concentration is a more dominant abiotic factor in nanoparticle dissolution. This is consistent with a previous study which noted that AgNP in Hoagland medium (pH 5.6), mimicking the ionic strength in riverine sediment, had lower rates of aggregation when the oxygen saturation of the medium was reduced [36]. Similarly, AgNP exhibited greater stability in anoxic and anaerobic freshwaters, with an increase of pH from 7.6 to more than 8.4 leading to a reduction of H^+^ concentration, which may have acted in parallel to reduce nanoparticle dissolution [37]. Decreases in pH were found to cause increased AgNP dissolution, although even at the lowest pH value of 4, oxygen was still shown to be a key factor that must be considered [28], particularly as dissolved oxygen may act to increase degree of aggregation; for example, oxygen saturated and unsaturated media showed aggregates of 3000 nm and 400–600 nm, respectively [38].

In the present study, under low oxygen saturation conditions, combinations of BSA-HA and HA-Alg resulted in increases of Ag^+^ ion concentrations of 10 and 19%, respectively, over 4 days at pH 6.6, while the corresponding samples at pH 7.8 showed increases of 5 and 15%, respectively. The BSA-Alg combination was found to stabilize the colloid against ion release more in the high pH water rather than low pH water, i.e., 3% ion concentration increase vs. 36%. As the salinity of the ASW was increased, the BSA-Alg NOM combination in all cases resulted in lower calculated Ag^+^ ion release than the corresponding BSA-HA and HA-Alg samples. For example, at pH 6.6 after 4 days, BSA-Alg samples had calculated Ag^+^ ion concentrations of 0.023 mg L^−1^, and 0.018–0.020 mg L^−1^ at pH 7.8. These values are much lower than the corresponding BSA-HA and HA-Alg values determined for pH 6.6 ASW (0.025–0.026 mg L^−1^ and 0.026 mg L^−1^, respectively) and pH 7.8 ASW (0.026 mg L^−1^ and 0.026 mg L^−1^, respectively). In summary, considering the data herein, changes in pH have been determined to impact on the dissolution process over a range of oxygen saturation levels that may be encountered in estuaries and shallow coastal waters, and the interaction of Alg with BSA-encapsulated AgNP was noted to provide the strongest stabilizing effect in terms of reducing Ag^+^ ion release at both high and low oxygen saturation levels. Overall, it is clear that pH and oxygen are important abiotic factors driving nanoparticle dissolution and modulate the stabilizing effect of different combinations of NOM.

**Figure 4:**
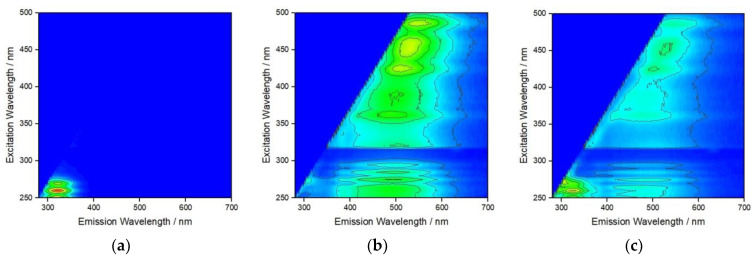
Fluorescence excitation–emission matrix for AgNP after addition of (**a**) BSA at S∙0, (**b**) BSA + HA at S∙10, and (**c**) BSA + HA + Alg at S∙38. (BSA, HA and Alg concentrations of 10, 10 and 1 mg L^−1^, respectively).

To probe the interaction of the different types of NOM that may sequentially encapsulate AgNP at different salinities as it transitions from fresh to estuarine, and eventually marine, waters fluorescence excitation–emission matrices were recorded (Figure 4). Strong fluorescence at an emission wavelength of about 320 nm was noted for BSA adsorbed to AgNP at S∙0 (Figure 4a), which gradually disappeared upon addition of HA and increase of salinity to S∙10 (Figure 4b). The emission at about 400–550 nm is ascribed to HA and covers a range of wavelengths due to the varied structure of HA and different environments of polyphenols present. This may indicate that, while a BSA coating on AgNP in saline water is highly stable in the absence of other NOM, HA disrupts and either replaces this coating, potentially resulting in protein salting out with consequent reduction in nanoparticle stability, or binds to BSA in the tryptophan environment in such way as to quench fluorescence [39]. The subsequent addition of Alg and the increase of salinity to S∙38 showed different behavior where emission from HA is still present, albeit with greatly reduced intensity, and a new emission is noted at about 320 nm, which is again assigned to BSA (Figure 4c). Fluorescence arising from Alg, based on the presence of residual polyphenols, could not be unambiguously assigned (Figure 4c). This may indicate that in a similar way, Alg may disrupt the previously formed HA coating on AgNP although not to the extent that the HA is completely replaced by Alg. As fluorescence from BSA has once again become visible, it is, therefore, reasonable to expect that BSA was not (fully) displaced from the nanoparticle surface after the initial addition of HA but rather non-radiative energy transfer (quenching) to polyphenol moieties of the HA may have masked the BSA fluorescence emission signal. Thus, the interplay between the hydrophobic domains of BSA, HA and Alg in the nanoparticle’s crowded near-surface region may be that which most influences the steric stabilization of AgNP in moving from estuarine to marine waters.

**Figure 5:**
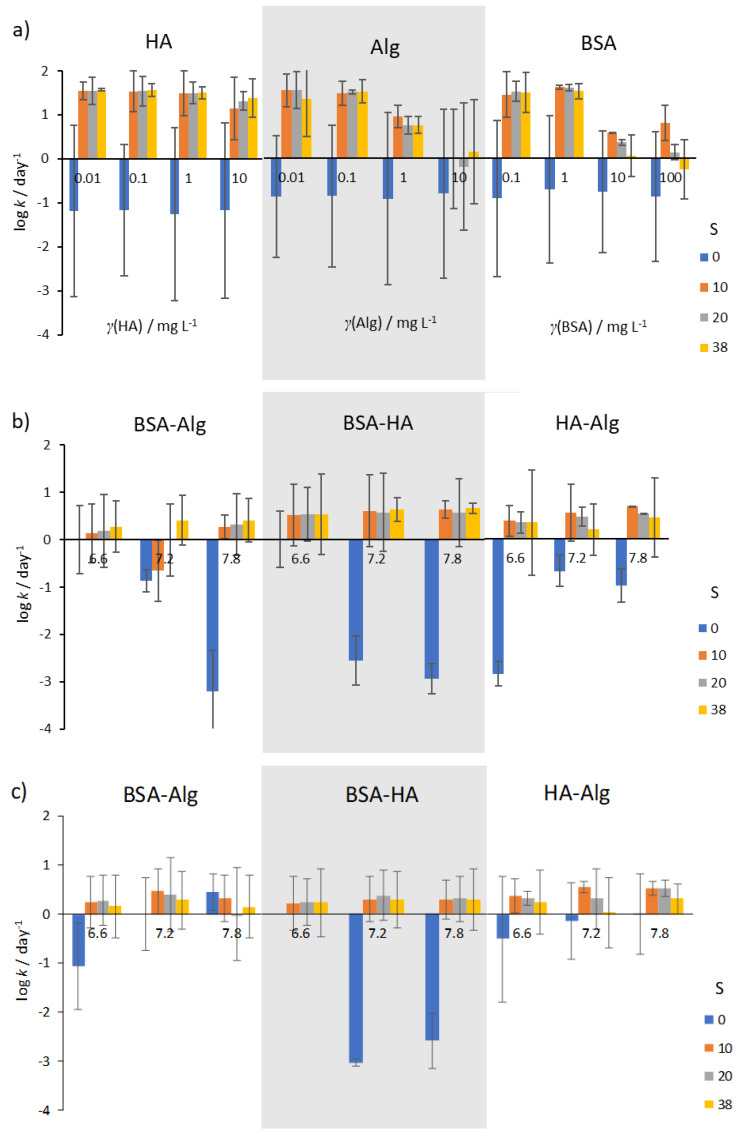
Kinetics of AgNP SPR absorption peak reduction in the presence of (**a**) NOM, (**b**) combinations of NOM at various pH (indicated under the bars), and (**c**) combinations of NOM at various pH and reduced O_2_ saturation (50%). Error bars indicate the standard deviation.

The SPR absorption peak of AgNP has previously been shown to be an excellent proxy for tracking the dissolution of 20 nm PVP-coated AgNP in ASW in place of ICP-MS, and with the added benefit of being a rapid and economical technique [40]. In the present work, a test of the linearity of the change of SPR absorbance with increasing Ag^+^ ion concentration, i.e., dissolution of AgNP, in ASW showed a good correlation (y = −0.0414x + 0.0273, R^2^ = 0.9392; Supplementary Figure S1) after 1–3 days. Sikder et al. [40] appropriately drew attention to the caveat that absorption coefficients must be determined for each medium in which AgNP dissolution is being tracked to allow the method to be used quantitatively, particularly if NOM is present. It must be further borne in mind that while SPR intensity can be a good proxy for Ag^+^ ion concentration in freshwaters [41], aggregation processes that occur in seawater also act to reduce the intensity of the SPR, therefore demonstrating that estimated Ag^+^ ion concentrations in the present work are only indicative (semi-quantitative). While developing precise tables of absorption coefficients is beyond the scope of the present work, light attenuation is recognized as an important variable to be considered when probing the impact of light intensity on NOM-facilitated silver reduction [13,32] in models of estuarine water. As water transparency is low in most large estuaries, any ionic silver deriving from AgNP would likely only undergo reduction in a shallow layer penetrable by UV light. Thus, dissolution is expected to remain the dominant process in estuaries. The kinetics of SPR reduction, as a semi-quantitative means of estimating the rate of AgNP dissolution, are shown in Figure 5 and Supplementary Table S1. Calculated Ag^+^ concentrations were plotted as a function of time and were modeled using the first order equation [28,38,42].

[Ag^+^]_t_ = [Ag^+^]_final_(1 − e^−*k*t^) (2)

where [Ag^+^]_t_ is the calculated concentration of Ag^+^ ions at time t, [Ag^+^]_final_ is the maximum concentration of nanoparticle-derived Ag^+^ ions as t→∞ and *k* is the rate coefficient of dissolution. It should be noted that the Ag^+^ concentration did not reach a plateau after 4 days, hence did not correspond to physicochemical ‘equilibrium’ in which the concentration of ions in solution is independent of the amount of solid present [42]. Thus, there remains a strong concentration gradient from the AgNP to the surrounding medium, with Cl^−^ quickly removing released Ag^+^. However, this is modulated by a second process in which released Ag^+^ may form a range of species, including AgCl_2_^−^, AgCl_3_^2−^ and AgCl_4_^3−^, at the nanoparticle surface which can acting as a passivating layer, slowing the further release of Ag^+^. Indeed, this effect has been shown to increase with ionic strength for 50 nm AgNP and may become more relevant as AgNP move from freshwater to brackish and seawater [43].

In well-oxygenated waters, rates of AgNP dissolution were found to vary with salinity. In mQ water (S∙0), irrespective of type or concentration of NOM, dissolution rates were low (typically about 0.1–1 day^−1^; Figure 5a), while dissolution was oftentimes more than two orders of magnitude greater in artificial seawater. HA was not found to reduce dissolution rate except at the highest concentration of 10 mg L^−1^, while Alg showed about 10 times slower dissolution at all salinities. Similarly, BSA was found to be effective at slowing dissolution rate at concentrations in the range 10–100 mg L^−1^.

Selecting concentrations of 10, 10 and 1 mg L^−1^ for BSA, HA and Alg, respectively, sequential treatment of AgNP with BSA and HA, or with BSA and Alg resulted in lower dissolution rates at all salinities and pH values (Figure 5b). In particular, AgNP coated by BSA followed by HA addition showed the lowest dissolution rates at pH 6.6, with rate of dissolution increasing with pH and salinity. However, the lowest dissolution rate for AgNP coated with BSA and Alg was noted at pH 7.8 in the highest-salinity water (S∙38), while reduction in nanoparticle dissolution was least for AgNP treated with the combination of HA and Alg. In saltwater with low oxygen saturation (Figure 5c), the rate of dissolution of AgNP was in most cases lower than in the corresponding oxygen-saturated media (Figure 5b).

This is in line with expectations that decreased oxygen concentration would impact the oxidative dissolution process such that release of Ag^+^ would occur at a slower rate. Interestingly, low oxygen saturation in mQ did not provide the expected enhanced stability in most nanoparticle dispersions. Irrespective of NOM, it may be expected that the diameter of AgNP (and hence surface area) would have a strong effect on dissolution.

However, the coefficient of dissolution for 5 nm AgNP (pH 5.7) [28] of 0.01 h^−1^ is similar to that for 20 nm and 40 nm AgNP (pH 5.6), with calculated values of 0.014 h^−1^ and 0.013 h^−1^, respectively [38]. For comparison, the dissociation constant of 0.010 h^−1^ (0.249 day^−1^) for our 40 nm AgNP at pH 6.6 is in line with these published data.

**Figure 6:**
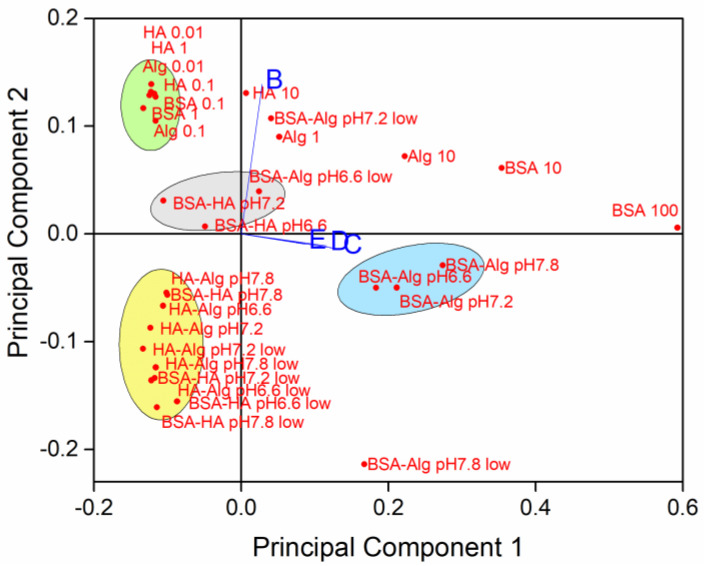
Principal components of sample variability based on NOM type and concentration (individual NOM given in mg L^−1^; pH 7.8), pH, oxygen saturation (low = 50% saturation) and salinity (B-E; S∙0, 10, 20 and 38, respectively).

To determine relationships between the simultaneously varying NOM type and concentration, salinity, pH and oxygen saturation level, principal component analysis was carried out on SPR absorption intensity with orthogonal ordination of the first two principal components, encompassing 94.33% of the variance, shown in the loading and scores bi-plot (Figure 6).

The loading plot indicates that data from dispersions at salinities S∙10, 20 and 38 (C, D and E, respectively) are correlated, while samples in mQ (S∙0; B) are relatively unrelated to the samples in saltwater. Several clusters of samples were noted, with AgNP coated with BSA followed by Alg showing the greatest effect on reducing nanoparticle dissolution in well oxygenated waters, irrespective of pH (Figure 6, blue ellipse). On the contrary, there was a slight negative correlation of BSA and HA coated AgNP with stabilization at lower pH. In freshwater, individual NOM tended to stabilize AgNP against dissolution (green ellipse), while combinations of NOM (with HA as one of the components), which is more likely to be encountered in the environment, enhanced dissolution of AgNP at all pH values and at oxygen saturation in the range 50–100% (yellow ellipse).

A range of reports focusing on the effects of NOM on AgNP have found that it can have both stabilizing and destabilizing properties. Studies of HA in freshwater have shown its ability to impart greater colloidal stability by electrostatic and steric interactions with silver nanoparticles. HA is typically present in aquatic matrices as high molecular weight unsaturated molecules which are rich in sulphur and nitrogen, which can, in addition to oxygenated groups, coordinate to form a corona around the nanoparticle surface and provide sufficient steric hindrance to help suppress agglomeration [44,45]. In freshwater, humic and fulvic acids have most commonly been shown to inhibit AgNPs dissolution under slightly acid conditions, e.g., stabilization of up to 24 h at pH 5.6 [28]. However, similarly, Nordic aquatic fulvic acid, at a similar pH 5.5, was not found to impact on AgNP dissolution [30].

Comparable HA stabilization effects due to surface adsorption have been noted over a wide pH range for other nanoparticles such as those of titanium dioxide [46]. The capacity of NOM to increase the colloidal stability of TiO_2_ was found to increase with decreasing pH and higher ionic strength. This, and the observation that higher NOM concentrations improved stability, may indicate an increase in hydrophobicity at the NOM-particle interface with enhanced affinity of the NOM for the nanoparticle surface. At high NOM concentrations of HA and Alg, charge inversion may promote stronger electrostatic repulsion, fragmenting formed aggregates and hence enhancing colloidal stability of TiO_2_ [47].

In contrast, coordination of various HA functional groups to AgNP has also been found to complex to Ag^+^ being released from the surface and may help to remove silver chloride species from the surface, thus enhancing the rate of oxidative dissolution at the surface [13]. In addition, the strong affinity of sulphur for silver may also enhance Ag^+^ release from AgNP through sulfidation and can give rise to a range of structures from core-shell to hollow spheres [48]. Indeed, that study showed that S-rich HA can be sufficiently strong as to complete sulfidation of 20 nm AgNP within 1 h. Furthermore, ionic strength also plays a key role, and it was found that Nordic aquatic fulvic acid did not provide the same benefit in stabilizing the colloid as the salinity increased, with significant Ag^+^ ion release occurring [30].

Alginate and polysaccharides have also been shown to enhance the stability of AgNP in salt water and likely perform a similar role to HA in increasing steric hindrance around the AgNP [43,49]. However, there is a limit to the degree of stabilization that can be achieved, and as salinity increases, compression and disruption of the electric double layer around the nanoparticles, particularly due to divalent cations, begins to dominate resulting in significant agglomeration and aggregation [50,51].

Studies to date have focused to a great degree on the influence of individual NOM or abiotic parameters on the stability of AgNP in terms of agglomeration and ion release. However, the aquatic environment, particularly for estuaries and brackish water, is much more complex as there are likely a range of different types of NOM simultaneously present and AgNP will likely already be encapsulated in an eco-corona before they reach fresh, brackish, and salt waters. Herein, and as previously reported, BSA as a model for an eco-corona showed excellent ability to stabilize AgNP at all salinities [19,52]. However, as the BSA-coated AgNP comes into contact with other NOM in the environment, interaction between the two types of NOM leads to a decrease in stability that is slightly pH and ionic strength dependent. HA was found to be less destabilizing at lower pH and salinity, while Alg enhanced stability and reduced Ag^+^ release at higher pH and salinity. Considering that these are the conditions most likely to be encountered as AgNP transitions from freshwater to brackish and marine water, it follows that this gradient in type of NOM present may stabilize AgNP (as pH and salinity changes) for a sufficient period that they may be able to move from fresh to brackish water without complete dissolution. Furthermore, the reduced dissolution rates of AgNP sequentially coated with multiple types of NOM may have important implications for the persistence of AgNP in the water column, and hence their potential toxicity towards a wide range of organisms. Indeed, the source of such toxicity remains a topic of some debate and, while previously ascribed exclusively to released Ag^+^ ions [53], there is some evidence of a nanoparticle size effect as a significant, or in cases even primary, factor that gives rise to AgNP toxicity in biota [54,55]. In addition, the coating on AgNP has also been shown to be an important aspect which must be considered when determining the source of deleterious effects in biota [56]. Hence, the present results add to the understanding of the behavior and fate of AgNP in estuarine waters while under the simultaneous influence of many and varying factors, and may be of use in understanding potential routes to toxicity in this environmental compartment. Future work should focus on the nature of interaction between the various types of NOM in the corona around the nanoparticle, particularly in terms of packing and electrosteric interactions, and how this interaction is modulated by abiotic factors such as ionic strength, pH, dissolved oxygen, and temperature.

## 4. Conclusions

This study reports on the impact of abiotic parameters and combinations of NOM on the physico-chemical behavior of AgNP in fresh, brackish and salt waters. All of these factors were varied stepwise so as to systematically uncover their effect on nanoparticle agglomeration and dissolution as a proxy for conditions experienced by nanoparticles as they transition from fresh to salt water environments in estuaries. The persistence of AgNP over a period of several days and the colloids’ stability under various conditions was monitored primarily using the AgNP surface plasmon resonance. While past research has typically focused on resolving the effects of abiotic parameters or individual types of NOM separately, the present study has attempted a more realistic approach where the effect of varying such parameters simultaneously is investigated. It was found that BSA, as a representative of wastewater proteinaceous matter, provided good stability to AgNP in freshwater. When protein-coated AgNP moves from wastewaters to surface waters, the introduction of humic substances in the form of HA, which produced a close coating around the initial BSA coating, did not provide good stabilization of the colloids at pH 6.6. Representing AgNP moving into brackish water, the salinity and pH were increased to S·10 and 7.2, respectively, and the SPR gradually lost intensity over 4 days indicating nanoparticle dissolution. However, the presence of Alg at medium and high salinities (S·38) and with increased pH (7.8) imparted better stability to the colloids than HA, and may have displaced and substituted HA from the BSA-coated AgNP. The protein coating seems to be key as the interaction of HA and Alg without BSA did not give stable colloids under various salinities and pH values. In waters that are less well oxygenated, the BSA–Alg interactions were again the most favorable for enhancing AgNP persistence, where nanoparticles oxidative dissolution decreased under conditions of low oxygen (50% oxygen saturation) and decreasing H^+^ ion concentrations (to pH 7.8). Overall, this study demonstrates that changes in the nature of NOM in estuaries is not only favorable, but necessary for enabling long-term persistence of AgNP. Specifically, HA was able to stabilize colloids at low salinity and pH though not in high-salinity and high-pH waters, while the opposite was true for Alg. Ultimately, it has been shown that the interaction of different types of NOM at the nanoparticle surface is dynamic and modulated by a combination of abiotic factors, and which has important consequences for nanoparticle persistence in environmental compartments. This multi-factor study is an initial step towards presenting a more realistic picture of the behavior and fate of nanoparticles in complex aquatic matrices and indicates that the NOM profile of an estuary along the salinity/pH gradient is the key factor that governs colloid stability and nanoparticle persistence as it transitions from fresh to salt water.

## Data Availability

The data presented in this study are available on request from the corresponding author.

## Supplementary Materials

The following supporting information can be downloaded at: https://www.mdpi.com/article/10.3390/nano12020296/s1, Figure S1: UV-vis absorption spectra for AgNP in ASW, Figure S2: UV-vis absorption spectra of AgNP in (a) mQ water, and in ASW (S·38) after 5 min incubation with (b) BSA (10 mg L^−1^), (c) HA (10 mg L^−1^), and (d) Alg (1 mg L^−1^), Figure S3: Temporal change of UV-vis absorption spectra of AgNP (5 mg L^−1^) with BSA (100 mg L^−1^) in ASW (S·38) over 4 days; Table S1: Calculated rates of silver ion (Ag^+^) release from AgNP.
