# Supplementary material for: Simultaneous Influence of Gradients in Natural Organic Matter and Abiotic Parameters on the Behavior of Silver Nanoparticles in the Transition Zone from Freshwater to Saltwater Environments
Source: Nanomaterials (Basel). 2022 Jan 17;12(2):296. doi: 10.3390/nano12020296 (PMC8778534; doi:10.3390/nano12020296)
Supplement: Supplementary file 1 [file nanomaterials-12-00296-s001.zip › nanomaterials-1466827-supplementary.pdf]

nanomaterials

MDPI

Supplementary Materials

Simultaneous Influence of Gradients in Natural Organic Matter and Abiotic Parameters on the Behavior of Silver Nanoparticles in the Transition Zone from Freshwater to Saltwater Environments

Ivana Čarapar, Lara Jurković, Dijana Pavičić-Hamer, Bojan Hamer and Daniel Mark Lyons *

Center for Marine Research, Ruđer Bošković Institute, G. Paliaga 5, 52210 Rovinj, Croatia; ivana.hazdovac@irb.hr (I.Č.); lara.jurkovic@irb.hr (L.J.); dpavicic@irb.hr (D.P.-H.); bhamer@irb.hr (B.H.)
* Correspondence: lyons@irb.hr; Tel.: +385-52-804725; Fax: +385-52-804780

Table S1. Calculated rates of silver ion (Ag^+) release from AgNP.

O₂ saturation<br>Coating	Salinity	pH	γ/mg L⁻¹	normal<br>$[Ag^+]_f$ / mg L⁻¹	normal<br>k / day⁻¹	normal<br>R^2	reduced<br>$[Ag^+]_f$ / mg L⁻¹	reduced<br>k / day⁻¹	reduced<br>R^2
HA	0	6.6	0.01	4.26386	0.06542	0.99491	0.71808	0.36448	0.9043
			0.1	3.00768	0.06837	0.99163	0.49861	0.25947	0.9784
			1	4.0046	0.0563	0.99535	0.47398	0.24319	0.981
			10	4.01156	0.06795	0.99599	0.58972	0.35993	0.9694
Alg	0	6.6	0.01	1.88644	0.13676	0.98757	0.62524	0.26701	0.94758
			0.1	2.20435	0.13862	0.99553	0.69834	0.1761	0.91886
			1	2.08107	0.1218	0.99907	0.1227	0.58013	0.89572
			10	1.94834	0.15732	0.99902	0.72305	0.17493	0.94757
BSA	0	6.6	0.1	2.06837	0.12506	0.99784	0.63172	0.58238	0.95044
			1	1.65528	0.20337	0.99713	2.41708	0.12474	0.9918
			10	1.60925	0.17866	0.98817	0.64408	0.49129	0.98106
			100	2.41714	0.13705	0.99127	0.73788	0.34573	0.96604
HA	10		0.01	4.72734	35.21259	0.99998	4.87819	27.36315	0.99995
			0.1	4.65024	34.20346	0.99999	4.75578	27.46644	0.99994
			1	4.60888	31.05399	0.99999	4.72369	26.21981	0.99991
			10	4.42123	13.86086	0.99985	4.56098	15.47325	0.99978
Alg	10		0.01	4.73616	35.25302	0.99999	4.75639	27.12472	0.99995
			0.1	4.53847	30.39042	0.99996	4.79897	26.66101	0.99995
			1	4.3324	8.99592	0.96795	4.29212	16.31648	0.98692
			10	4.62939	0.97709	0.99534	4.35643	4.6009	0.95333
BSA	10		0.1	4.61709	28.04514	0.99998	4.89036	24.93495	0.99996
			1	4.68233	41.26388	0.99999	4.71183	27.65161	0.99998
			10	1.93378	3.80545	0.98461	2.12601	5.13404	0.9547
			100	0.82329	6.39518	0.89183	1.09851	0.28589	0.82499
HA	20		0.01	4.75532	35.29339	0.99999	4.91043	24.15264	0.99999
			0.1	4.67108	34.50134	0.99999	4.77433	24.43112	1
			1	4.64173	31.21376	0.99996	4.74589	23.57208	0.99997
			10	3.90511	20.66442	0.99963	4.10995	18.16369	0.99972
Alg	20		0.01	4.7608	35.50314	0.99999	4.77296	24.41726	0.99998
			0.1	4.51642	32.39285	0.99989	4.81524	23.4764	0.99997
			1	4.42514	5.66634	0.95438	3.88812	8.32747	0.90985
			10	4.75427	0.6499	0.99709	4.71273	0.64554	0.98121
BSA	20		0.1	4.65496	32.91209	0.99997	4.90815	23.3393	0.99998

			1	4.67062	39.55747	0.99996	4.67746	31.32767	0.99994
			10	2.67844	2.34583	0.93722	3.21959	9.14689	0.95602
			100	0.99907	1.38794	0.80148	1.06456	0.42426	0.85536
HA	38	7.8	0.01	4.76946	36.83918	0.99996	4.92321	31.73585	0.99999
			0.1	4.68355	36.15017	0.99998	4.78057	31.5457	0.99999
			1	4.63352	31.65206	0.99994	4.73373	27.75967	0.99994
			10	4.46761	23.83701	0.99993	3.99665	20.92694	0.9718
Alg	38	7.8	0.01	4.75441	22.43439	0.99999	4.78589	30.66561	1
			0.1	4.55763	33.2791	0.99998	4.80991	28.5747	0.99998
			1	4.42026	5.71489	0.95773	4.30664	6.19438	0.94581
			10	4.72963	1.42608	0.99883	4.77509	1.27076	0.99415
BSA	38	7.8	0.1	4.68415	31.50738	0.99998	4.92549	27.58061	0.99998
			1	4.63488	33.52187	0.99981	4.68917	29.15859	0.99986
			10	4.19977	1.14442	0.91675	4.12729	9.7064	0.93995
			100	1.77517	0.56304	0.77829	1.48696	0.44778	0.89767
BSA-Alg	0	6.6	10–1	0.02557	0.61973	0.99985	1.86167	0.08721	0.99965
		7.2		0.13971	0.13572	0.99993	106.894	6.55E−04	0.99949
		7.8		36.10179	6.22E−04	0.99995	1.82914	2.82202	0.99958
BSA-HA	0	6.6	10–10	0.02153	0.49209	0.99995	0.15984	3.06E−01	0.99995
		7.2		29.26054	0.0014	0.9998	34.53339	4.28E−04	0.99946
		7.8		41.64207	5.31E−04	0.99993	41.43086	0.00134	0.99977
HA-Alg	0	6.6	10–1	186.3103	6.29E−04	0.99891	0.96049	2.69E−01	0.99997
		7.2		0.36778	1.72E−01	0.99962	0.60519	6.92E−01	0.9998
		7.8		0.86263	0.07875	0.99942	0.49419	1.02481	0.99994
BSA-Alg	10	6.6	10–1	0.90203	1.37246	0.9995	3.51889	1.7786	0.99763
		7.2		0.33581	0.21995	0.99988	3.10131	2.91338	0.99953
		7.8		0.48714	1.84551	0.9997	1.78769	2.09997	0.99905
BSA-HA	10	6.6	10–10	2.69476	3.71309	0.99997	1.8096	1.75	0.99902
		7.2		3.42527	4.67439	1	1.79082	2.11698	0.99921
		7.8		2.45954	4.98157	0.99998	1.68286	2.09574	0.99902
HA-Alg	10	6.6	10–1	2.88231	2.61434	0.99888	2.51553	2.63	0.99908
		7.2		2.43551	4.1608	0.99999	2.65855	4.07949	0.9998
		7.8		2.88472	5.70106	0.99998	2.48407	3.95385	0.99979
BSA-Alg	20	6.6	10–1	1.84725	1.5566	0.9993	3.31599	1.88799	0.99798
		7.2		1.94802	0.98236	0.99778	4.62748	2.50908	0.99978
		7.8		1.50485	2.07958	0.99968	1.60819	0.97712	0.99931
BSA-HA	20	6.6	10–10	3.22649	3.88845	0.99997	1.74442	1.84022	0.9988
		7.2		3.65715	4.25127	0.99999	2.0541	2.62143	0.99969
		7.8		2.69144	4.23637	0.99999	1.79146	2.18254	0.99934
HA-Alg	20	6.6	10–1	2.8368	2.3985	0.99733	2.29093	2.34084	0.99616
		7.2		2.45024	3.29516	0.99948	2.63079	2.2775	0.99957
		7.8		2.71351	3.97114	0.99953	2.32367	3.94754	0.99904
BSA-Alg	38	6.6	10–1	2.68056	1.87196	0.99831	3.49785	1.43084	0.99718
		7.2		2.38819	2.55819	0.9995	3.67633	1.93955	0.99871
		7.8		2.47729	2.55363	0.99928	1.88892	1.41292	0.99838
BSA-HA	38	6.6	10–10	3.43427	3.82612	0.99999	2.03303	1.79615	0.99948
		7.2		3.66484	5.09963	0.99998	1.84078	2.13164	0.99955
		7.8		2.80625	5.35569	0.99998	1.72506	2.05725	0.99964
HA-Alg	38	6.6	10–1	2.82934	2.41614	0.99997	2.38428	1.8825	0.99939
		7.2		2.49552	1.6385	0.99816	2.83763	1.09793	0.99763
		7.8		2.56386	3.13236	0.99997	2.28678	2.23551	0.99801

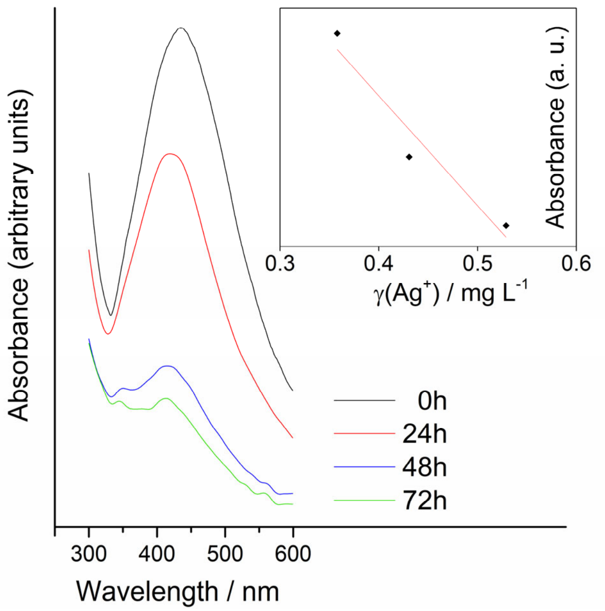

Figure S1. UV-vis absorption spectra for AgNP in ASW. Inset: Temporal change in surface plasmon resonance peak maximum as a function of measured Ag^+ concentration after 24, 48 and 72 hours. Linear fit $y = -0.0414x + 0.0273$, $R^2 = 0.9392$.

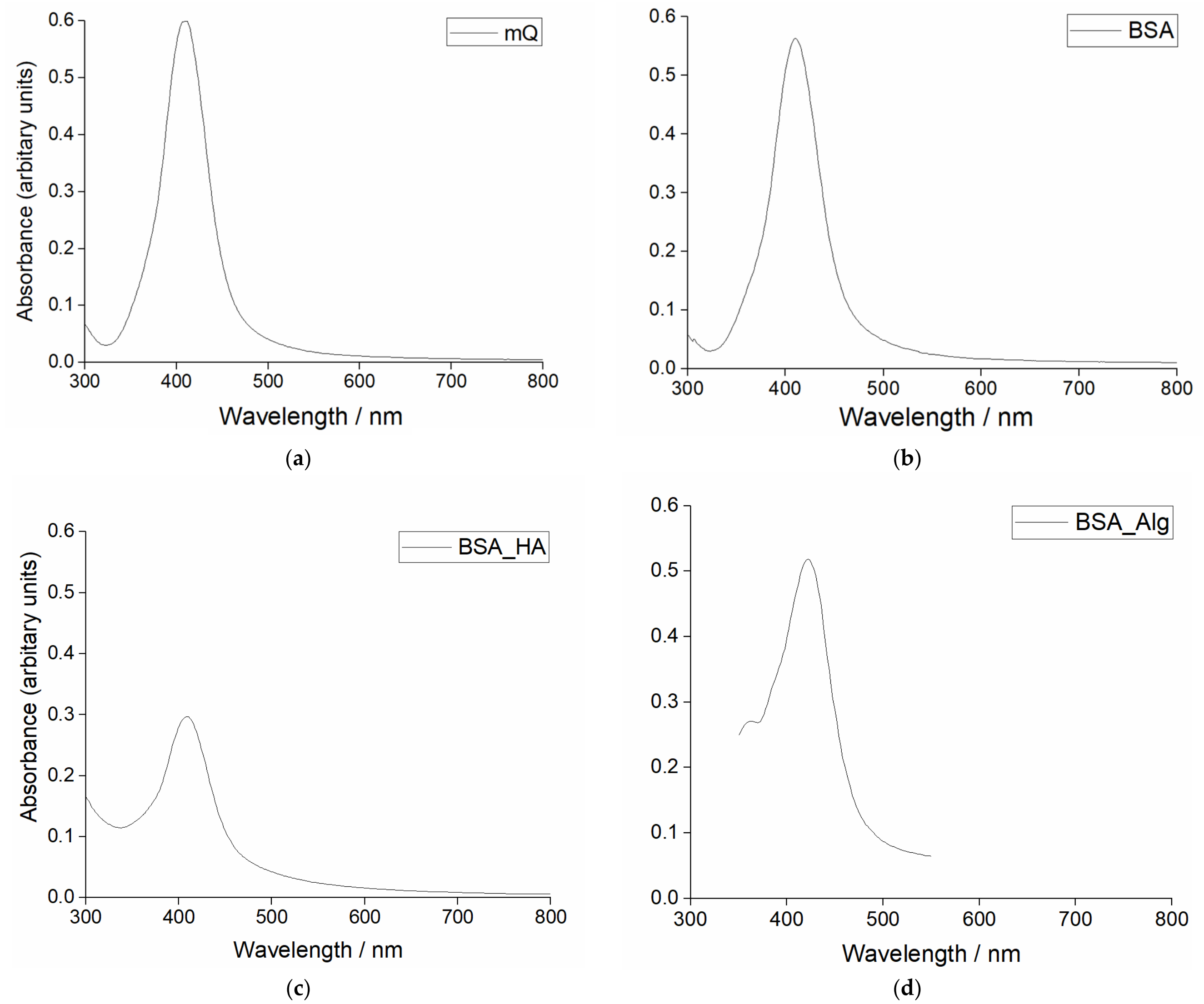

Figure S2. UV-vis absorption spectra of AgNP in (**a**) mQ water, and in ASW (S·38) after 5 min incubation with (**b**) BSA (10 mg L^{-1}), (**c**) HA (10 mg L^{-1}), and (**d**) Alg (1 mg L^{-1}).

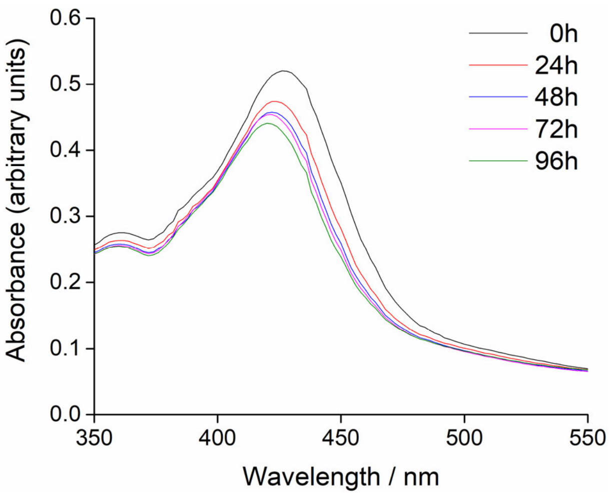

Figure S3. Temporal change of UV-vis absorption spectra of AgNP (5 mg L^{-1}) with BSA (100 mg L^{-1}) in ASW (S·38) over 4 days.
